# The Alantic Monthly

**Published:** 1863-10

**Authors:** 


*The Alantic Monthly.*—This most popular of American periodicals, is placed upon our table by the favor of the publishers, Messrs. TICKNOR & FIELDS, Boston. The October number contains articles from Charles Sumner, John G. Whittier, R. W. Emerson, H. D. Thoreau, J. W. Trowbridge, J. P. Quincy, D. A. Wasson, F. D. Hedge, C. C. Hazewell, G. B. Prescott, and others. While it is announced that the November number will contain contributions in prose and verse, from Henry W. Longfellow, James Russell Lowell, Oliver Wendell Holmes, Professor Agassiz, Louisa M. Alcott, J. K. Marvel, T. B. Aldrich, H. D. Thoreau, and other well known writers.

The eleventh volume of the "Atlantic," comprising January to July, 1863, is now neatly bound in muslin. No periodical can better deserve the popularity it enjoys. It should be placed upon the table of every family who read English.

